# Design of a microwave spectrometer for high-precision Lamb shift spectroscopy of antihydrogen atoms

**DOI:** 10.1007/s10751-024-01876-3

**Published:** 2024-03-01

**Authors:** T. A. Tanaka, P. Blumer, G. Janka, B. Ohayon, C. Regenfus, M. Asari, R. Tsukida, T. Higuchi, K. S. Tanaka, P. Crivelli, N. Kuroda

**Affiliations:** 1 Institute of Physics, The University of Tokyo, Komaba, Meguro-ku, 153-8902 Tokyo Japan; 2 Institute for Particle Physics and Astrophysics, ETH Zürich, Otto-Stern-Weg, Zürich, 8093 Switzerland; 3 The Helen Diller Quantum Center, Department of Physics, Technion-Israel Institute of Technology, Haifa, 3200003 Israel; 4 Research Center for Nuclear Physics (RCNP), Osaka University, Mihogaoka, Ibaraki-shi, 567-0047 Osaka, Japan; 5 Research Institute for Science and Engineering (RISE), Waseda University, Okubo, Shinjuku-ku, 169-0072 Tokyo Japan; 6 Present Address: Laboratory for Muon Spin Spectroscopy, Paul Scherrer Institute, Forschungsstrasse, 5232 Villigen PSI, Switzerland; 7 Present Address: Institute for Integrated Radiation and Nuclear Science (KURNS), Kyoto University, Asashiro-nishi, Kumatori-cho, 590-0494 Osaka, Japan

**Keywords:** Microwave spectroscopy, Lamb shift, Antihydrogen, Antiproton, Charge radius

## Abstract

We have developed a microwave spectrometer for a measurement of the $\varvec{2S_{1/2}-2P_{1/2}}$ Lamb shift of antihydrogen atoms towards the determination of the antiproton charge radius. The spectrometer consists of two consecutive apparatuses, of which the first apparatus, *Hyperfine Selector* (HFS), filters out $\varvec{2S_{1/2}(F=1)}$ hyperfine states and pre-selects the $\varvec{2S_{1/2}(F=0)}$ state, and the second apparatus, *MicroWave Scanner* (MWS), sweeps the frequency around the target transition to obtain the spectrum. We optimized the geometry of the apparatuses by evaluating the S-parameter that represents the ratio of the reflected microwave signal over the input, utilizing microwave simulations based on the finite element method. The HFS was designed to obtain a resonant property at 1.1 GHz for an efficient removal of the $\varvec{2S_{1/2}(F=1)}$ hyperfine states, and the MWS was designed to realize weak frequency-dependency in the signal reflection. Also, the spatial distributions of microwave electric field were simulated. We report the design of the spectrometer and discuss an expected precision of the first measurement.

## Introduction

The proton charge radius (PCR) was experimentally determined for the first time in 1955 by an elastic scattering experiment to inject an electron beam to a gaseous hydrogen target [1]. Up to the present date, the PCR has been precisely measured by a variety of methods such as laser or microwave spectroscopy of hydrogen atoms, as well as by electron scattering experiments.

In 2010, high-precision laser spectroscopy of muonic hydrogen atoms — the bound state of a proton and a muon, whose mass is 207 times heavier than that of the electron — was achieved to find a smaller PCR value of 0.84184(67) fm [2] than the CODATA value of 0.8768(69) fm at the time [3]. This $5.0\,\sigma$ disagreement marked the beginning of the so-called PCR puzzle and was followed by further experiments. For example, Lamb shift spectroscopy of hydrogen atoms by E. A. Hessels and co-workers found a PCR value agreeing with the smaller value [4]. See recent reviews [5] for more information on the progress of both the experimental and theoretical works.

Meanwhile, the experimental determination of the antiproton ($\bar{p}$) charge radius has not yet been carried out. In recent years, however, there have been significant technical advances in experiments producing antihydrogen ($\bar{\textrm{H}}$) —the simplest anti-atomic system consisting of an $\bar{p}$ and a positron ($e^+$) — in the Antiproton Decelerator (AD) / ELENA facility at CERN. For stringent tests of Charge-Parity-Time (CPT) symmetry, magnetic trapping and high-precision laser and microwave (MW) spectroscopy of $\bar{\textrm{H}}$ atoms have been achieved [6–10]. In addition, the Weak Equivalence Principle on $\bar{\textrm{H}}$ atoms was tested by measuring the gravitational acceleration between $\bar{\textrm{H}}$ atoms and the Earth [11].

The GBAR (Gravitational Behaviour of Anti-hydrogen at Rest) experiment running in the AD / ELENA facility aims at a precise measurement of the free fall acceleration of $\bar{\textrm{H}}$ atoms using antihydrogen ions ($\bar{\textrm{H}}{^{+}}$) produced by the following two-stage charge-exchange reactions with positronium ($\textrm{Ps}$) atoms — a bound state of $e^-$ and $e^+$ [12, 13]:

1 $$\begin{aligned} \bar{p} + \textrm{Ps} \longrightarrow \bar{\textrm{H}} + e^- \end{aligned}$$

2 $$\begin{aligned} \bar{\textrm{H}} + \textrm{Ps} \longrightarrow \bar{\textrm{H}}{^+} + e^- \end{aligned}$$

They have recently succeeded in producing beam-like $\bar{\textrm{H}}$ atoms [14], using ground state $\textrm{Ps}$ atoms at the first stage of the reactions. Under this production scheme, calculations predict the presence of 2*S* state $\bar{\textrm{H}}$ atoms [15].

In this context, we aim at the first measurement of the $\bar{p}$ charge radius by high-precision spectroscopy of $2S_{1/2}-2P_{1/2}$ Lamb shift transition using the $\bar{\textrm{H}}$ atomic beam [16, 17]. In this article, we describe the experimental method and the design of the MW spectrometer to be used in the Lamb shift spectroscopy, as well as the expected precision.

## Experimental method

### Spectroscopy setup in the $\bar{\textrm{H}}$ beam line

**Fig. 1:**
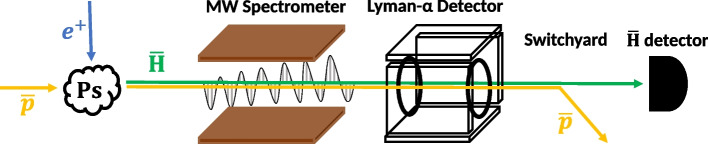
Schematic view of the $2S_{1/2}-2P_{1/2}$ Lamb shift spectroscopy experiment

A schematic view of the $\bar{\textrm{H}}$ $2S_{1/2}-2P_{1/2}$ Lamb shift spectroscopy experiment is shown in Fig. 1. The trajectories of $\bar{p}$, $e^+$, and $\bar{\textrm{H}}$ are represented by orange, blue, and green arrows, respectively. The $\textrm{Ps}$ production target is depicted as a cloud. The 2*S* state $\bar{\textrm{H}}$ (lifetime of 0.12 s) first passes through the MW spectrometer, which irradiates a MW electric field (MW E-field) in the range of 0.6 GHz to 1.5 GHz. If the Lamb shift transition is induced, the 2*S* states are de-excited to the 2*P* states (lifetime of 1.6 ns), which are promptly followed by the Lyman–$\alpha$ transition to the ground state. After the spectrometer, the number of remaining 2*S* states is counted by applying a DC electric field (DC E-field), inducing the de-excitation to the ground state through the Lyman–$\alpha$ transition via Stark mixing. The Lyman–$\alpha$ detector downstream to the MW spectrometer (Fig. 1) is developed for this purpose, which consists of two ring electrodes to apply the DC E-field and CsI coated microchannel plates (MCPs) [18] to detect the Lyman–$\alpha$ photons. By repeating this procedure with different MW frequencies for each pulse of $\bar{\textrm{H}}$ beam, the Lamb shift resonance spectrum will be obtained as a decrease in the Lyman–$\alpha$ photon counts.

After the spectroscopy region, an electrostatic switchyard and a $\bar{\textrm{H}}$ detector are located. The switchyard deflects $\bar{p}$ and only the $\bar{\textrm{H}}$ beam arrives at the $\bar{\textrm{H}}$ detector downstream [14]. The $\bar{\textrm{H}}$ detector comprises a MCP, a phosphor screen, and a CCD camera. $\bar{\textrm{H}}$ detection here will be used for a coincidence analysis of the Lyman–$\alpha$ detector.

### Apparatuses for the high-precision spectroscopy

Under CPT symmetry, the $2S_{1/2}$ and $2P_{1/2}$ states of an $\bar{\textrm{H}}$ atom should have the same hyperfine structure levels $F=0$ and 1 (where *F* represents the total angular momentum) as those of a $\textrm{H}$ atom. With respect to $\textrm{H}$ atoms, the transition frequency between the $2S_{1/2}$ $F=0$ and $F=1$ level is measured to be 177.5568343(67) MHz [19], and the transition frequency between the $2P_{1/2}$ $F=0$ and $F=1$ level is reported to be 59.22(14) MHz [20] experimentally and 59.1695(6) MHz theoretically [21].

**Fig. 2:**
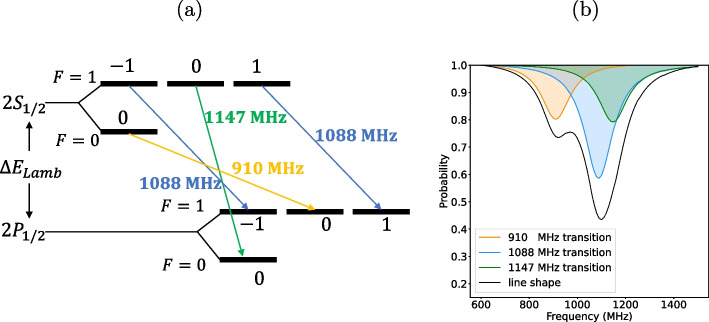
(a) Scheme of the $2S_{1/2}-2P_{1/2}$ Lamb shift transition and (b) observable line shape with the decomposed contributions of the three transitions

As illustrated in Fig. 2(a), the Lamb shift transition by a linearly polarized MW E-field is composed of three transitions originating from the hyperfine structures of both the $2S_{1/2}$ and $2P_{1/2}$ states. The transition frequencies, without being affected by any external fields, are about 910 MHz, 1088 MHz, and 1147 MHz. Because the lifetime of 2*P* states is relatively short (1.6 ns), each resonance line shape of these transitions has a natural width of about 100 MHz, and mutually overlaps. As a result, the observable line shape of $2S_{1/2}-2P_{1/2}$ Lamb shift transition appears as the black solid line in Fig. 2(b), where the contributions of the three transitions are represented by orange, blue, and green filled area. For the transitions of 1088 MHz and 1147 MHz, the resonant frequency is too close for each peak to be distinguishable, and only a single peak around 1.1 GHz is observed, which makes it difficult to precisely determine the transition frequencies.

**Fig. 3:**
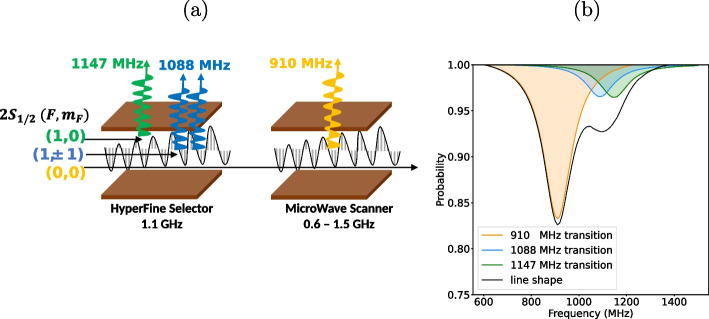
(a) Schematic view of the spectroscopy setup consisting of two consecutive MW apparatuses, and (b) observable line shape with the decomposed contributions, when the HFS is utilized

To handle this problem, a setup consisting of multiple MW apparatuses were developed in the history of hydrogen Lamb shift experiments [4, 22, 23]. In our case, the setup consisting of two consecutive MW apparatuses is adopted as shown in Fig. 3(a). The first apparatus *Hyperfine Selector* (HFS) filters out the $2S_{1/2}$ $F=1$ states beforehand by irradiating a MW field at frequency of 1.1 GHz to simplify the resonance. The second apparatus *Microwave Scanner* (MWS) sweeps the MW frequency in the range of 0.6 GHz to 1.5 GHz to obtain a spectrum with a single peak dominantly made by the pre-selected $2S_{1/2}$ $F=0$ state. Consequently, the line shape of the transition appears as the black line in Fig. 3(b), where the contributions of the 1088 MHz transition and 1147 MHz transition are suppressed.

**Fig. 4:**
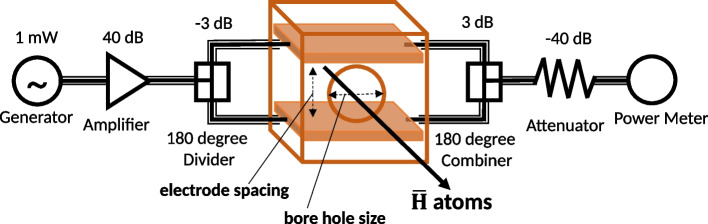
Schematic view of the MW apparatus and associated components of the MW circuit

A 50 $\Omega$ impedance matched transmission line type MW apparatus was developed [24], which is composed of a pair of parallel plate electrodes and a rectangular box, based on a hydrogen Lamb shift experiment [23]. Between each electrode of the pair, a MW E-field perpendicular to the beam axis can be formed, whose spatial distribution is uniform even for a beam with a relatively large radial size. The MW apparatus and the associated MW circuit components are schematically illustrated in Fig. 4.

The MW signal, first generated by a high-frequency signal generator at 1 mW, is amplified to 10 W by a 40 dB amplifier, and then split by a 180 degree phase inverting high power divider. Inside the MW apparatus, each of the phase inverted signals is fed to the upper and the lower electrode via a SMA connector fixed on the rectangular box with its pin stuck into the electrode, forming a constructively oscillating MW E-field between the electrodes. Then, the signal is transmitted through to the SMA connectors on the other side. After the MW apparatuses, the signal is combined, attenuated, and the power is measured at the end, in order to estimate the frequency-dependency of the MW field amplitude during the spectroscopy. The entire MW circuit, including coaxial cables, consists of 50 $\Omega$ impedance-matched products.

Using the MWS and HFS of this structure with the electrode spacing and bore hole size of 20 mm, high-precision Lamb shift spectroscopy of muonium atoms were achieved [24–26].

In the case of the $\bar{\textrm{H}}$ beam line in the GBAR experiment, the set of MW apparatuses with 30 mm bore hole size is favored not to interrupt the $\bar{\textrm{H}}$ atoms and $\bar{p}$ beam. In the following, optimization of the geometric parameters of these MW apparatuses is reported, based on MW simulations.

## Geometric optimization of the microwave apparatuses

The different purpose of the two MW apparatuses leads to indivisual optimization strategies.

Regarding the MWS, which is scanned over the frequency range of 0.6 GHz to 1.5 GHz, it is preferred that the MW E-field amplitude has the least dependence on the frequency. Therefore, we look for such geometry that enables the apparatus to function as a 50 $\Omega$ impedance matched transmission line, to suppress the frequency-dependency of the reflection of input MW signal over the frequency range.

Regarding the HFS, on the other hand, which is operated at a fixed frequency of 1.1 GHz, it is preferred that the MW E-field amplitude at this frequency is maximized to efficiently remove the $2S_{1/2}$ $F=1$ states. Therefore, we look for such geometry that enables the apparatus to resonate at an input frequency of 1.1 GHz, to gain the MW power inside the apparatus.

**Fig. 5:**
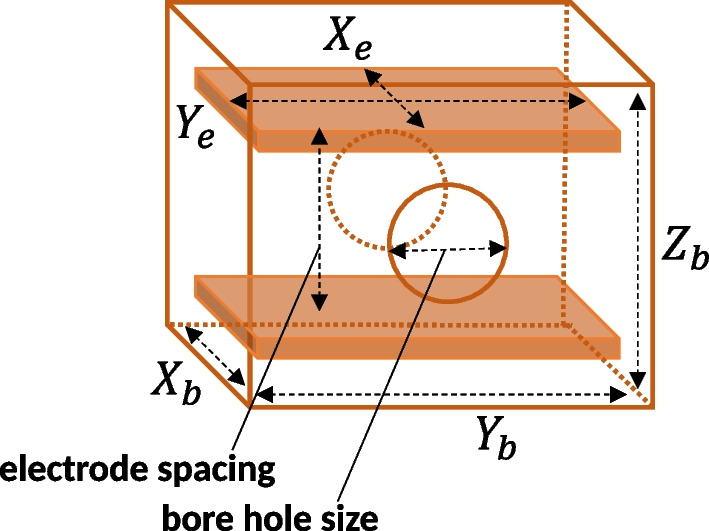
Schematic view of the geometric parameters targeted to optimize by MW simulations

Figure 5 shows major geometric parameters of the MW apparatus. The parameters $X_b$, $Y_b$ and $Z_b$, stand for the internal measure of the box, along the $\bar{\textrm{H}}$ beam direction (x-axis), the horizontal direction (y-axis), and the vertical direction (z-axis) respectively. The $X_e$ and $Y_e$ stand for the lengths of the electrode in each respective direction. The electrode spacing and bore hole size are kept to be 30 mm for both the MWS and HFS.

CST Studio Suite 2021 (Microwave Studio) https://www.cst.com/ was used for MW simulations. To optimize the geometric parameters, the S-parameter $S_{11}$, which represents the ratio of the reflected MW signal over the input, was calculated for different geometries.

### Optimization of the MWS

Several sequences of the parameter sweep simulation were performed for optimizing the geometry of the MWS, and $X_b$, $X_e$, $Y_b$, $Y_e$, and $Z_b$ were determined to be 40, 26, 100, 98, and 60 mm respectively, with a consideration for mechanical constraints due to our chamber size.

The MWS is assembled from copper plates to form the electrodes and rectangular box. The parameter $Z_b$ is adjustable from 50 mm to 60 mm for tuning the MW transmission property.

**Fig. 6:**
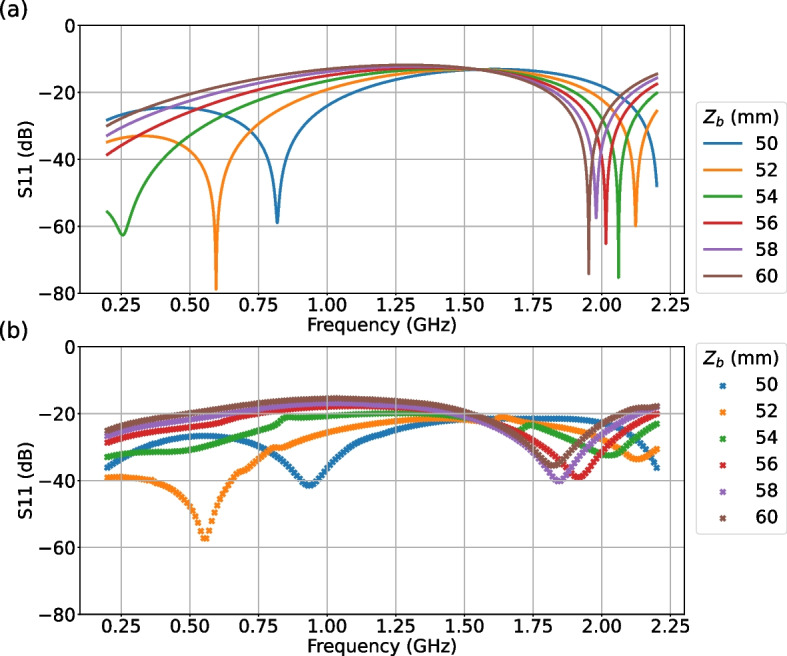
(a) Simulated and (b) measured $S_{11}$ of the MWS, for different values of $Z_b$

The $S_{11}$ of the manufactured MWS was measured using a Vector Network Analyzer (Keysight E5061B). Figure 6 shows the simulated and measured $S_{11}$ obtained for different values of $Z_b$. One can see that the $S_{11}$ is suppressed to be less than –10 dB over the entire frequency range. Also, it is indicated that one can avoid the dip of $S_{11}$ around 0.6 GHz by adopting higher values of $Z_b$, to reduce the frequency-dependency around the Lamb shift transition frequency.

The discrepancy of each resonant peak position of simulated and measured $S_{11}$ in Fig. 6, which is about 150 MHz at maximum for $Z_b$ $=50$, is considered to be caused by the assembly accuracy against the geometry of CAD model used in simulations. This can be circumvented by adjusting $Z_b$. For the adjustment of $Z_b$, the spacing between top plate and bottom plate can be tuned by changing the number of washers used for screws to form the rectangular box from the copper plates.

### Optimization of the HFS

The parameter sweep simulations were performed for the HFS as well, and it was found that a resonant property for the HFS is implemented by changing the geometry of rectangular box while fixing that of electrodes.

**Fig. 7:**
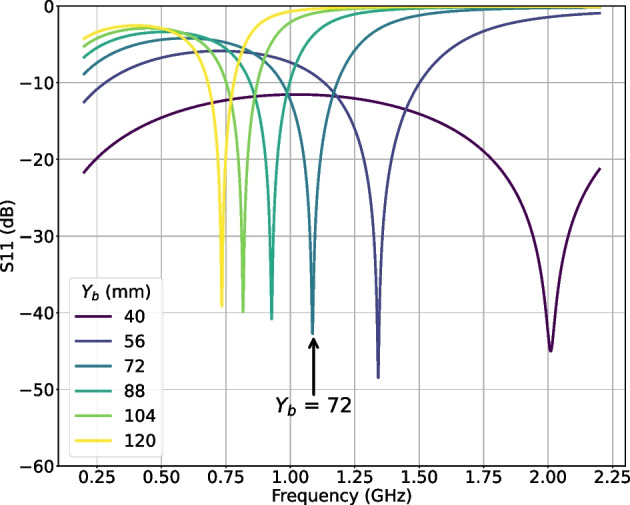
Simulated $S_{11}$ of HFS, for different values of $Y_b$

Figure 7 shows the simulated $S_{11}$ for different values of $Y_b$, when $Y_e$ is fixed to be 32 mm. The $X_b$ and $X_e$ are the same values as those of the MWS. The $Z_b$ is set 100 mm, which is the maximum value under the constraints for mechanical realization, in order to gain E-field flux in between the electrodes rather than from the electrode to the rectangular box.

As seen in Fig. 7, the resonant peak position moves to lower frequency and the baseline gets higher with increasing $Y_b$. This indicates that the resonant property starts to show up for the frequency of the peak position, and Q value rises up accordingly. To implement the resonant property at 1.1 GHz, the geometry with $Y_b$ around 72 mm seems suitable, as annotated in Fig. 7.

The HFS is assembled from copper plates to form the electrodes and rectangular box, and copper lines to connect the electrodes to the SMA connectors. The parameter $Y_b$ is adjustable by a few mm around 72 mm.

**Fig. 8:**
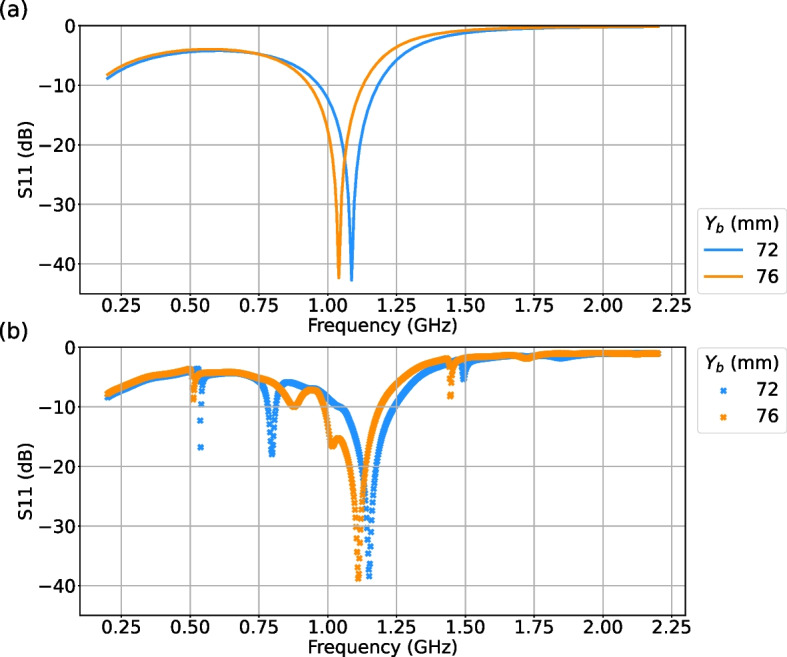
(a) Simulated and (b) measured $S_{11}$ of the HFS, for different values of $Y_b$

The $S_{11}$ was measured for this HFS as well. In Fig. 8 the simulated and the measured $S_{11}$ are compared for two different values of $Z_b$ $=72$ and 76 mm. For both geometries, one can see a dip around 1.1 GHz and a good agreement in the overall shape of the spectra between the simulations and measurements.

## Spatial distribution of the microwave field

In order to characterize the spatial distribution of the MW E-field, the field map was calculated using CST Studio. The MWS is expected to form an E-field distribution with a small frequency-dependency, whereas the E-field formed by the HFS is expected to be maximized at 1.1 GHz as a result of the resonant property.

**Fig. 9:**
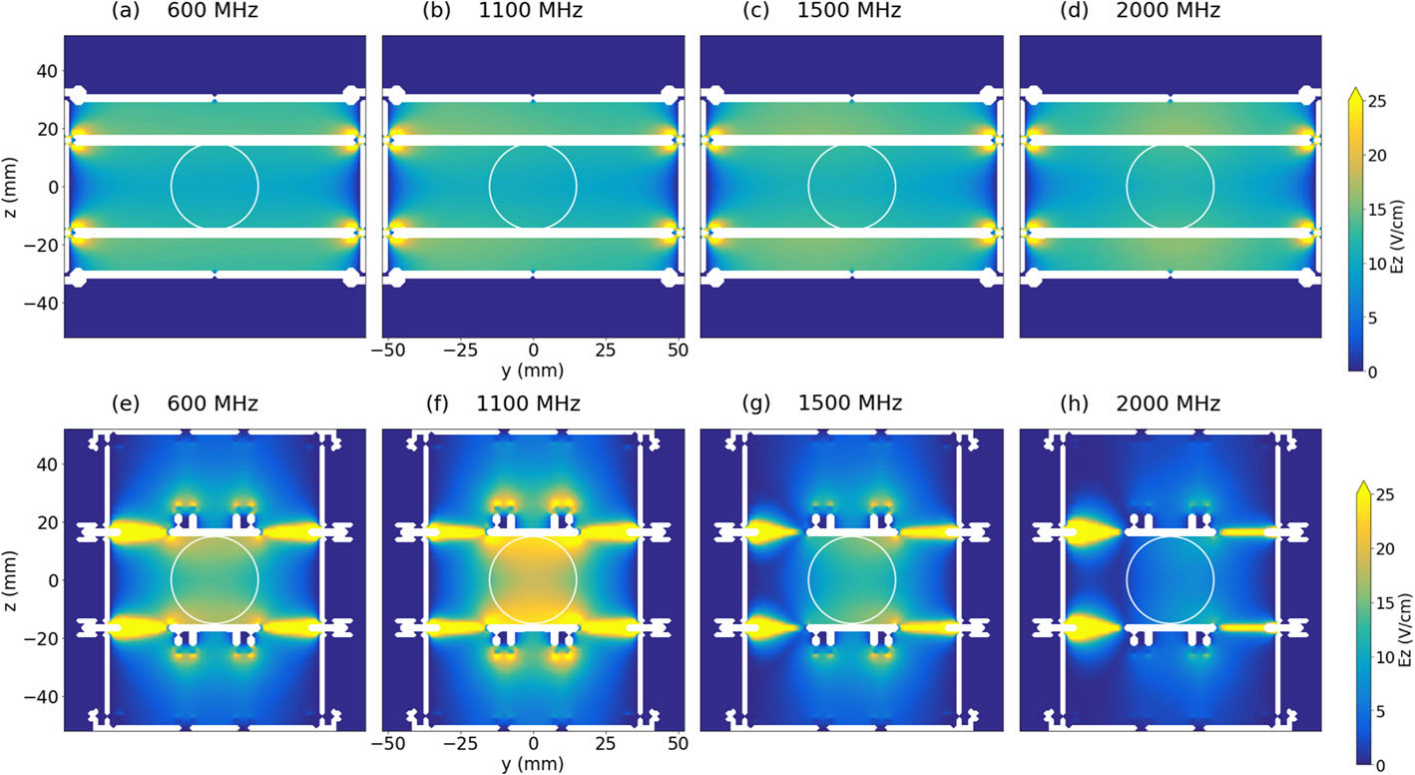
2D field map of time averaged $E_z$ formed by the MWS at an input MW power of 10 W, cut by the yz plane. The maps are drawn for different frequencies of (a) 600, (b) 1100, (c) 1500, and (d) 2000 MHz, from the left to the right. The same series of field maps (e-h) are drawn for the HFS

Figure 9 shows the 2D field map of time averaged $E_z$ formed by the MWS at an input MW power of 10 W, cut by the yz plane. The maps are drawn for different frequencies of (a) 600, (b) 1100, (c) 1500, and (d) 2000 MHz, from the left to the right. Figure 9(e-h) is the same series of field maps about $E_z$ formed by the HFS.

One can see almost identical distributions for the MWS, regardless of the variation of frequency. This supports its usage as a MW transmission line for this range of input frequency.

On the other hand, in the case of the HFS, the maps are relatively frequency-dependent. The overall distribution is the highest at 1100 MHz as seen in Fig. 9(f). This agrees with the resonant peak in $S_{11}$, in the sense that the input MW signal is not reflected but fully transmitted inside. Also, the asymmetric distribution at 2000 MHz seen in Fig. 9(h) implies that the majority of the input MW signal fed from the left side by the two SMA connectors is reflected, which agrees with the high $S_{11}$ of almost 0 dB around this frequency.

Around the coordinate origin, $E_z$ formed by the HFS at 1100 MHz is about 60% higher than that by the MWS, even with the same input MW power. This is considered to be resulting from the resonant property of the HFS around this frequency.

## Monte-Carlo simulation

A Monte-Carlo simulation has been developed for the spectroscopy setup in the $\bar{\textrm{H}}$ beam line. In this simulation, a 3D grid data of the MW E-field formed by the HFS at 1100 MHz and MWS for different frequencies, and a 3D grid data of the DC E-field formed by the Lyman–$\alpha$ detector are imported from CST Studio. While the $\bar{\textrm{H}}$ atoms follow the trajectories in the simulation, the time development of probability coefficient from each state is calculated by numerically solving the Schrödinger equation, using the E-field amplitude at the nearest grid for each step.

**Fig. 10:**
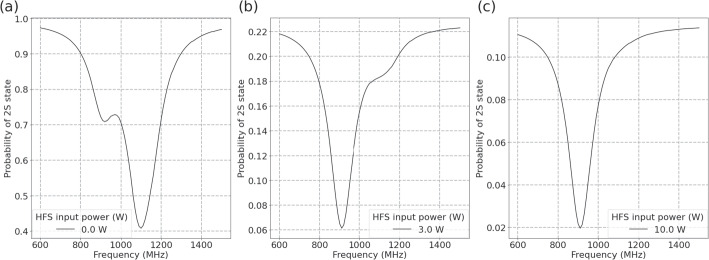
Observable line shapes when the HFS is fed with (a) 0 W, (b) 3 W, and (c) 10 W

This simulation has been utilized to evaluate a necessary input MW power for the apparatuses to induce the Lamb shift transitions. For example, the state selection efficiency of the HFS can be studied. Figure 10 shows the observable line shapes when an on-axis beam interacts with the MW E-fields, at different input powers of (a) 0 W, (b) 3 W, and (c) 10 W for the HFS. One can see that 10 W is sufficient to remove the contribution of $2S_{1/2}$ $(F=1)$ state $\bar{\textrm{H}}$ atoms.

It is also noted in Fig. 10 that the probability coefficient of $2S_{1/2}$ state, or the event rate of detecting Lyman–$\alpha$ photons downstream, diminishes with higher input MW power. As such, the optimization of the input MW power will be studied using this simulation to resolve the trade-off between measurement precision and time.

Also, this simulation helps to estimate the number of events needed for achieving a given precision of the spectroscopy. In the planned experiment, $\bar{\textrm{H}}$ detection is one of the event selection criteria. Hence, in the preliminary stage of the simulation, the $\bar{\textrm{H}}$ atoms are ejected from a point source located at the exit of $\textrm{Ps}$ target, and only the $\bar{\textrm{H}}$ atoms hitting the MCP of the $\bar{\textrm{H}}$ detector are studied.

To estimate the experimental precision, Lyman–$\alpha$ photon emission is also simulated, based on the change in probability of 2*S* states at the beginning and end of a step. To assess the efficiency of the Lyman–$\alpha$ detection, the channel opening and the quantum efficiency of the CsI-coated MCPs are taken into account [24].

Currently, with an assumption that about 100 $2S_{1/2}$ state $\bar{\textrm{H}}$ atoms are produced per pulse beam of $\bar{p}$ from ELENA operating with duty cycle of 80%, about 3 weeks of data taking is expected to achieve a precision of 1200 ppm. This will update the record by the precedent measurement [10].

## Summary and outlook

The MW spectrometer was developed for the spectroscopy of the $2S_{1/2}-2P_{1/2}$ Lamb shift transition of $\bar{\textrm{H}}$ atoms, which can determine the antiproton charge radius. The geometric structure of the HFS was optimized to obtain a resonant property at 1100 MHz to remove the $2S_{1/2}$ $F=1$ states efficiently. In contrast, the MWS was optimized to suppress the MW signal reflection to be flat and low.

The time averaged amplitude of the MW E-fields were investigated to characterize the spatial distribution of the E-field formed by the MW apparatuses. We confirm that the HFS forms an E-field of the highest intensity at the resonant frequency of 1.1 GHz, and the MWS forms a relatively homogeneous E-field regardless of the variation of frequency, as expected from the design based on the behaviour of S-parameters.

Also, a numerical calculation code was developed including the spatial distribution of the MW E-field. Using an on-axis pencil beam, the state selection efficiency of the HFS was confirmed to be almost complete at an input MW power of 10 W. For estimating the precision in the planned spectroscopy, the calculation code also includes the DC E-field formed by the Lyman–$\alpha$ detector, and takes into account the detection efficiency of the Lyman–$\alpha$ photons to perform a Monte-Carlo simulation. From the simulation we can deduce that a few weeks of data taking is promising to determine the Lamb shift frequency at an order of 1000 ppm.

The commissioning of the MW spectrometer is ongoing in the GBAR experiment. $2S_{1/2}$ state $\textrm{H}$ atoms can be produced by the $\mathrm {H^-}$ ion beam from ELENA impinging on a thin carbon foil. Spectroscopic studies and the Lyman–$\alpha$ photon detection will be demonstrated with a $\textrm{H}$ atomic beam.

A first detection of Lyman–$\alpha$ photons from $2S_{1/2}$ state $\bar{\textrm{H}}$ atoms is planned for end of 2024 with an increasing precision on the Lamb shift transition over the next years, depending on the available number of produced $\bar{\textrm{H}}$ atoms in the GBAR experiment.

## Data Availability

The data and simulation code are available from the corresponding author upon reasonable request.

## References

[1] Hofstadter, R., McAllister, R.W.: Phys. Rev. **98**, 217 (1955)

[2] Pohl, R., et al.: Nature **466**, 213–216 (2010) 20613837 10.1038/nature09250

[3] Mohr, P.J., Taylor, B.N., Newell, D.B.: Rev. Mod. Phys. **80**, 633–670 (2008) 10.1103/RevModPhys.93.025010 PMC9890581 36733295

[4] Bezginov, N., Valdez, T., Horbatsch, M., et al.: Science **365**, 1007 (2019) 31488684 10.1126/science.aau7807

[5] Gao, H., Vanderhaeghen, M.: Rev. Mod. Phys. **94**, 015002 (2022)

[6] Ahmadi, M., et al.: Nature **548**, 66–69 (2017) 28770838 10.1038/nature23446

[7] Ahmadi, M., et al.: Nature **541**, 506–510 (2017) 28005057 10.1038/nature21040

[8] Ahmadi, M., et al.: Nature **557**, 71–75 (2018) 29618820 10.1038/s41586-018-0017-2 PMC6784861

[9] Ahmadi, M., et al.: Nature **561**, 211–215 (2018) 30135588 10.1038/s41586-018-0435-1 PMC6786973

[10] Ahmadi, M., et al.: Nature **578**, 375–380 (2020) 32076225 10.1038/s41586-020-2006-5 PMC7162817

[11] Anderson, E.K., et al.: Nature **621**, 716–722 (2023) 37758891 10.1038/s41586-023-06527-1 PMC10533407

[12] The GBAR collaboration, CERN Report No. CERN/SPSC-P-342 (2011)

[13] Perez, P., et al.: Hyperfine Interact. **233**, 21 (2015)

[14] Adrich, P., et al.: Eur. Phys. J. C **83**, 1004 (2023)

[15] Rawlins, C.M., Kadyrov, A.S., Stelbovics, A.T., Bray, I.: Phys. Rev. A **93**, 012709 (2016)

[16] Crivelli, P., et al.: Phys. Rev. D **94**, 052008 (2016)

[17] Kuroda, N., et al.: J. Phys. Conf. Ser. **875**, 022054 (2017)

[18] Janka, G., Ohayon, B., et al.: Eur. Phys. J. C **80**, 804 (2020) 32922165 10.1140/epjc/s10052-020-8400-1 PMC7462919

[19] Kolachevsky, N., Matveev, A., Alnis, J., et al.: Phys. Rev. Lett. **102**, 213002 (2009) 19519101 10.1103/PhysRevLett.102.213002

[20] Lundeen, S., Jessop, P., Pipkin, F.: Phys. Rev. Lett. **34**, 377 (1975)

[21] Kramida, A.: At. Data and Nucl. Data Tables **96**(6), 586–644 (2010)

[22] Newton, G., et al.: Phil. Trans. R. Soc. A **290**, 1373 (1979)

[23] Lundeen, S.R., Pipkin, F.M.: Metrologia **22**, 9 (1986)

[24] Janka, G.: Ph.D. Thesis, ETH Zürich (2022)

[25] Ohayon, B., et al.: Phys. Rev. Lett. **128**, 011802 (2022) 35061492 10.1103/PhysRevLett.128.011802

[26] Janka, G., et al.: Nat. Commun. **13**, 7273 (2022) 36433948 10.1038/s41467-022-34672-0 PMC9700798

